# Mental Health Recovery of Evacuees and Residents from the Fukushima Daiichi Nuclear Power Plant Accident after Seven Years—Contribution of Social Network and a Desirable Lifestyle

**DOI:** 10.3390/ijerph15112381

**Published:** 2018-10-27

**Authors:** Masatsugu Orui, Satomi Nakajima, Yui Takebayashi, Akiko Ito, Maho Momoi, Masaharu Maeda, Seiji Yasumura, Hitoshi Ohto

**Affiliations:** 1 Department of Public Health, Fukushima Medical University School of Medicine, Fukushima 960-1295, Japan; yasumura@fmu.ac.jp; 2 Faculty of Human Studies, Musashino University, Tokyo 135-8181, Japan; satonaka@musashino-u.ac.jp; 3 Radiation Medical Science Center for the Fukushima Health Management Survey, Fukushima Medical University, Fukushima 960-1295, Japan; takeb-ky@fmu.ac.jp (Y.T.); itoaki@fmu.ac.jp (A.I.); maho-m@fmu.ac.jp (M.M.); masagen@fmu.ac.jp (M.M.); hit-ohto@fmu.ac.jp (H.O.); 4 Department of Disaster Psychiatry, Fukushima Medical University School of Medicine, Fukushima 960-1295, Japan

**Keywords:** mental health, nuclear disaster, great East Japan earthquake, recovery, social network, social role, desirable lifestyle

## Abstract

The 2011 Fukushima nuclear accident resulted in the exposure to radiation and evacuation, which has created psychological distress among the Fukushima residents. With the provision of multi-faceted support and the progress of the reconstruction, their mental health has appeared to show signs of recovery. However, there have been few studies investigating their recovery. To clarify the related factors associated with mental health recovery, a cross-sectional questionnaire survey was conducted. Subjects whose answers were associated with Resilience, Recovery, and Remitting patterns of mental health status were categorized in the Recovery group, while those associated with Delayed/Chronic dysfunction were placed in the Non-recovered group. In a multivariable logistic regression analysis, disaster-related unemployment (odds ratio (OR): 0.80, 95% CI (confidence interval): 0.65–0.99) and economic hardship (OR: 0.80, 95% CI: 0.65–0.98) were associated with the hindrance of recovery. In contrast, overall good health (OR: 1.47, 95% CI: 1.20–1.80), regular physical activity (OR: 1.23, 95% CI: 1.01–1.50), social interaction with friends (OR: 1.25, 95% CI: 1.00–1.55), and established social roles (OR: 1.44, 95% CI: 1.14–1.82) were associated with the promotion of recovery. In conclusion, our study showed a positive association between mental health recovery and a desirable lifestyle and social network, particularly with social roles. Thus, the provision of active social roles can promote recovery related to a disaster as with multi-faceted support.

## 1. Introduction

The Great East Japan Earthquake, which occurred on 11 March 2011, was the largest earthquake ever recorded in Japan’s history. The earthquake (magnitude 9.0) generated a massive tsunami that caused enormous damage to the Pacific Coast. This was followed by a separate tsunami, which hit the Fukushima Daiichi Nuclear Power Plant operated by the Tokyo Electric Power Company, and caused a radiation disaster in the Fukushima Prefecture that required the long-term evacuation of residents from many surrounding municipalities. As of May 2016, more than 92,000 residents who lived near the nuclear power plant had been forced to leave their homes at the directive of the Japanese government due to this triple disaster [1].

The earthquake and nuclear accident caused multiple psychological reactions among the evacuees and residents of Fukushima which included traumatic responses [2,3,4], loss of family, relatives, and friends [5], and the perceived health risk due to radiation exposure [6,7]. Under these circumstances, the suicide rate in Fukushima increased after 2–3 years of the disaster occurrence [8,9]. In addition to other mental health problems, in particular, the perceived radiation exposure risk, may have resulted in a prejudiced attitude among the public, or chronic anxiety among the evacuees. These effects could cause sociopsychological issues such as public or self-stigma [9,10]. Their mental health status had also been affected by loss of employment and/or community ties due to the nuclear disaster and residential relocation with consequent drastic changes in their living circumstances [11].

Despite this harsh situation, there has been gradual progress in the reconstruction of Fukushima after the nuclear disaster in the past seven years since the accident [12]. Although the mental health status among some evacuees and residents appeared to be recovering due to frequent opportunities for support or aid in the post-disaster period [13,14,15,16,17,18] and reconstruction progress [19,20], there have been few studies investigating mental health recovery from the Fukushima nuclear disaster. We hypothesized that desirable lifestyle or adequate social networks could promote mental health recovery. Therefore, the present study aimed to clarify the related factors associated with the recovery of mental health status such as desirable lifestyle or adequate social networks among the evacuees and residents by conducting a cross-sectional questionnaire survey in Fukushima. These findings will most likely be useful for future disaster risk reduction and management of mental health problems.

## 2. Materials and Methods

### 2.1. Participants

**Figure 1:**
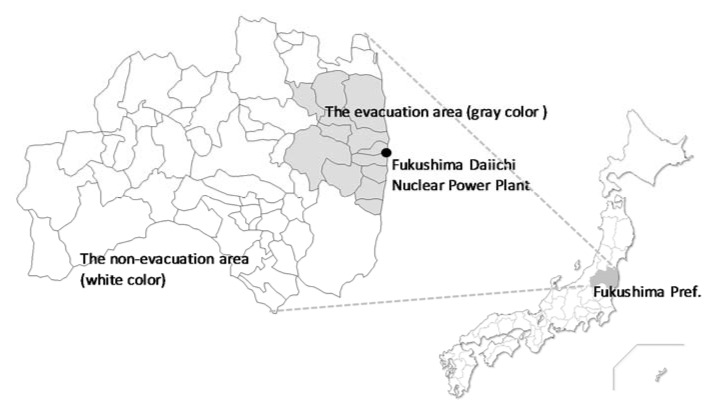
Location of the Fukushima Daiichi Nuclear Power Plant and the evacuation/non-evacuation areas. The gray color shows the evacuation area and the white color shows the non-evacuation area (as of December 2015).

This cross-sectional questionnaire survey targeted 1000 residents of the Fukushima Prefecture aged 20 and above. We selected 500 people from the evacuation area comprising Tamura City, Minami-Soma City, Kawamata Town, Hirono Town, Naraha Town, Tomioka Town, Okuma Town, Futaba Town, Namie Town, Kawauchi Village, Katsurao Village, and Iitate Village. The Japanese government has designated evacuation areas according to the spatial radiation dose rates as follows: (1) difficult-to-return areas with a radiation dose rate ≥50 millisieverts (mSv) per year; (2) residence restricted areas with a radiation dose rate ≥20 and <50 mSv per year; and (3) areas where evacuation orders were ready to be lifted as of 22 April 2011. Those living in the evacuation area were forced to leave their homes at the direction of the Japanese government. Five hundred people in the non-evacuation area were selected (Figure 1) using a two-stage stratified random sampling (stage one comprising a regional survey, and stage two comprising an individual survey) method. Thirty to 35 individuals per area were randomly selected from municipal resident registration files to obtain 1000 representative participants. We sent an anonymous, self-reporting postal questionnaire to participants from January to February 2018. The survey was approved by the ethics review committee of Fukushima Medical University on 10 October 2017 (No. 29206).

### 2.2. Survey Variables

For related factors to mental health recovery, (1) disaster-related experiences; (2) economic status; (3) general health status and lifestyle, and (4) social network (social interacting with friends from pre-disaster, places to communicate about the disaster, and social roles through daily activities) were set as dependent variables.

#### 2.2.1. Disaster-Related Experiences

Needless to say, disaster-related experiences such as evacuation, separation from family members, housing damage, and loss of family, relatives or friends are associated with mental health status [5,21]. The effects of the disaster on socioeconomic statuses such as the loss of employment was also hypothesized as a risk factor for mental health recovery [22]. Disaster-related experiences including evacuation, separation from family members, housing damage (severe/partial collapse), loss of family, relatives or friends, and disaster-related loss of employment were evaluated on a two-point scale defined as “Experienced” or “Never”.

#### 2.2.2. Economic Status

It has been reported that socioeconomic circumstances may affect evacuees’ psychological status [23]. Thus, in order to evaluate the relationship with the mental health recovery, economic status was assessed by the following question “Do you feel that you can afford your current economic status?” and answered on the five-point scale “Difficult”, “Somewhat difficult”, “Average”, “Somewhat enough”, and “Enough”.

#### 2.2.3. Subjective Health Status and Lifestyle

Subjective health status in general health status and lifestyle were scored based on a five-point scale ranging from “Very well”, “Well”, “Unremarkable”, “Poor”, and “Very poor”. To evaluate lifestyle that might be related to mental health recovery, we investigated sleep satisfaction and changes in physical activity after the disaster [24,25]. Sleep satisfaction was assessed on a four-point scale ranging from “Really satisfied”,” Satisfied”, “Dissatisfied”, and “Really dissatisfied”. Change in physical activity level was recorded as “Increase”, “No change”, and “Decrease” when compared to the pre-disaster level. Some disaster studies have reported that alcohol consumption increased following a disaster, due to psychological distress in the affected individuals [26,27], therefore, a change in alcohol consumption was assessed as “Increase”, “No change”, “Decrease”, or “Non-drinker” when compared with the pre-disaster level in order to assess the relationship with mental health recovery. As a previous study reported that laughter may lower the risk of poor subjective health [28], laughing frequency was asked on a four-point scale ranging from “Almost every day”, “1–5 times per week”, “1–3 times per month”, and “Never or almost never” based on the previous study [29].

#### 2.2.4. Social Network

Social networks were considered as an important factor influencing mental health outcomes, and high social capital played an important role in protecting mental health [30]. Therefore, we assessed the association between mental health recovery and social networks by utilizing the following three questions: (1) Social interaction with friends from pre-disaster, “Have you been interacting with friends from pre-disaster?”; (2) Places to communicate about the disaster, “Do you have places where you feel free to talk about the disaster?”; and (3) Social roles through daily activities, “Do you feel that you are helpful to others through your job(s), housework, or social activities?”. Participants answered these social network questions on a five-point scale ranging from “Strongly agree”, “Agree”, “Neutral”, “Disagree”, and “Strongly disagree”. These responses were categorized into two groups: “Agree” and “Disagree/Neutral” while analyzing the related factors with mental health recovery.

#### 2.2.5. Mental Health Recovery Patterns

**Figure 2:**
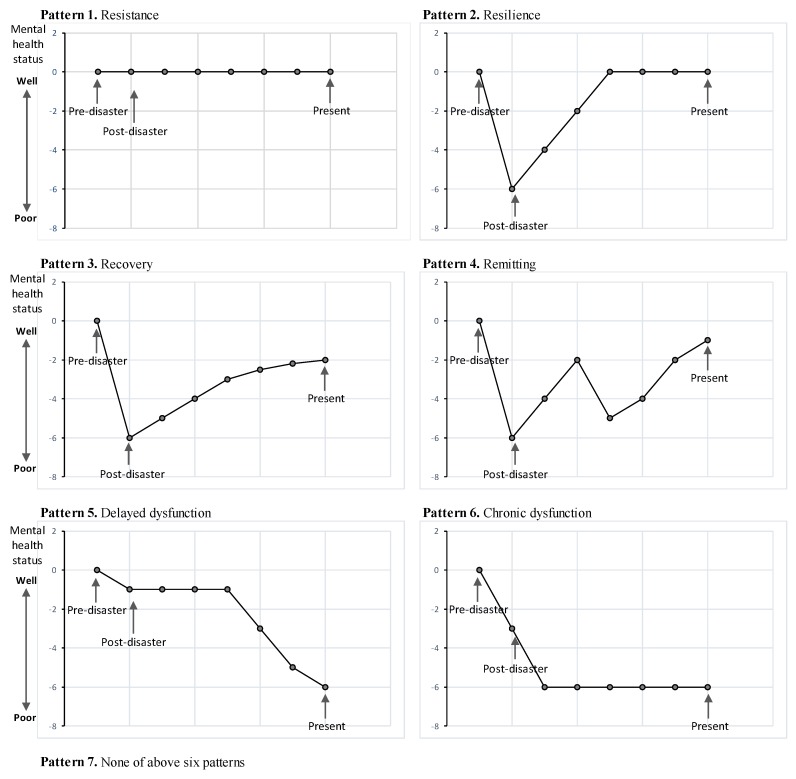
The mental health recovery patterns: the trajectory models of resilience, recovery, remitting, and delayed/chronic dysfunction presented by Norris et al. (2009) [31] was utilized as the measurement scale for our questionnaire. Participants were asked the subjective question of which of the seven patterns most appropriately described their mental health status changes from pre-disaster to its current state and they selected the most appropriate choice from six mental health recovery patterns.

We used the trajectory models of resistance, resilience, recovery and delayed/chronic dysfunction presented by Norris et al. (2009) to assess the independent variables associated with the recovery of mental health status [31]. Participants were asked the subjective question “Which of the seven patterns most appropriately describe your mental health status changes from pre-disaster to its current state? (1) Resistance; (2) Resilience; (3) Recovery; (4) Remitting; (5) Chronic dysfunction; (6) Delayed dysfunction; and (7) none of the six patterns” and to select the most appropriate choice (Figure 2).

To assess the mental health status in the “Recovered” and “Non-recovered” groups, we utilized the K6 scale to screen for non-specific psychological distress [32]. Those scoring 0–12 points were classified as having probable mild–moderate/probable no psychological distress and those scoring 13–24 points were classified as having probable severe psychological distress [32]. This study used the Japanese version of the K6, which has been empirically validated as an independent means of screening for psychological distress among evacuees [33].

### 2.3. Statistical Analysis

The chi-square test and multivariable logistic regression models were used to examine the association of mental health recovery with disaster-related experiences, general health status, and lifestyle, economic status, and social network variables. In particular, two models were analyzed in multivariable logistic regression analysis: Model 1 was adjusted by age, gender, disaster-related experiences, and current economic status, which was observed to affect disaster-associated mental health recovery [30,34,35]; and Model 2 had current health status and lifestyle (general subjective health status, sleep quality, physical activity level, and laughing frequency) and social network status added to the variables in Model 1, which could be protective for their mental health [24,25,28,36,37,38,39], and evacuees received health guidance as a part of disaster health activities [40,41,42]. Moreover, the *t*-test was utilized to assess the K6 scale in the “Recovered” and “Non-recovered” groups.

Statistical significance was evaluated using two-sided, design-based tests with a 5% level of significance. All statistical analyses were performed using SPSS 24.0 (IBM Corp., Armonk, NY, USA).

## 3. Results

### 3.1. Participants

**Figure 3:**
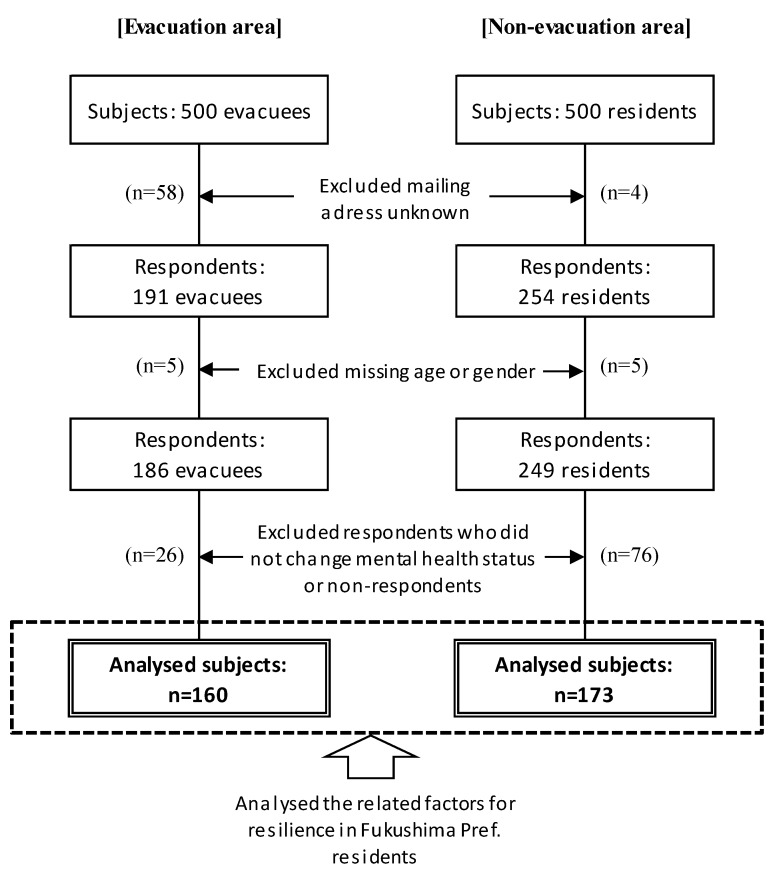
Sample selection in the evacuation and non-evacuation area: Among the 1000 subjects, 191 evacuees in the evacuation area and 254 residents in the non-evacuation area responded to the questionnaire. After excluding respondents who were missing age and gender information, and who did not change their mental health status or non-respondents, we analyzed 160 subjects in the evacuation area and 173 subjects in the non-evacuation area.

We sent out 938 questionnaires (excluding 62 subjects that were returned to the sender as no one was residing at the address), and received 445 responses (response rate, 47.4%). After excluding 10 respondents who failed to provide information regarding age or gender, and 102 respondents who answered “Resistant” and “None of previous six patterns” of the mental health recovery pattern or did not answer this question, the final study population was comprised of 233 respondents (Figure 3).

### 3.2. Respondent Characteristics

Table 1 shows the characteristic of each mental health recovery pattern. In those with a “Resistance” pattern, there was the tendency of a higher proportion of younger subjects, male, employed, and living in the non-evacuation area on 11 March 2011. Whereas, in those with a “Chronic dysfunction” pattern, there was the tendency of a higher proportion of older subjects, unemployed, and living in the evacuation area as of 11 March 2011. The mean K6 score among total cases was 6.0 while those of Delayed/Chronic dysfunction were higher than other patterns (relatively 11.8 and 12.3).

Based on the response of the mental health recovery patterns, we defined those who selected the “Resilience”, “Recovery”, and “Remitting” patterns into the “Recovered group” and those who selected the “Delayed dysfunction” and “Chronic dysfunction” pattern into the “Non-recovered group”. In this study, those who selected “Resistance” or “None of six patterns” were excluded from analysis because their mental health status between pre- and post-disaster was unchanged or unknown. There were 274 subjects in the “Recovered group” and 59 in the “Non-recovered group”. There were 80.0% in the “Recovered group” in the evacuation area 80.0%, and 84.4% in the non-evacuation area, which was not significantly different. There was a higher proportion of unemployed respondents in the “Non-recovered group” compared to the “Recovered group”. The proportion of those with a K6 score ≥ 13 was significantly higher in the “Non-recovered group” than in the “Recovered group” (Recovered: 32.5%, Non-recovered: 67.5%), with a corresponding significantly higher mean K6 score in the non-recovered group (Recovered: 4.81 point, Non-recovered: 12.1 point) (Table 2).

### 3.3. Disaster-Related Experiences and Current Economic Status

Table 3 shows the distribution of disaster-related experiences and current economic status in the recovered and non-recovered groups. Loss of family, relatives and friends, disaster-associated loss of employment, and higher current psychological distress were significantly high in the “Non-recovered group”. In contrast, experiences of evacuation, disaster-associated separation of family members, and house damage were not significantly associated with mental health recovery in this study. Economic hardship was significantly associated with non-recovered mental health.

### 3.4. Current Health Status, Lifestyle, and Social Network

The current health status, lifestyle, and social network among the “Recovered” and “Non-recovered group” are shown in Table 4. Poor general subjective health status, a dissatisfactory sleep condition, decreased physical activity level, and lower laughing frequency were significantly associated with non-recovered mental health. In contrast, social interacting with friends from pre-disaster and social roles through daily activities were associated with mental health recovery.

### 3.5. Association between Mental Health Recovery and Disaster-Related Experience, Current Economic Status and Health Status, and Lifestyle and Social Network

In the multivariable logistic regression analysis, Model 1 was adjusted for age, gender, disaster-related experiences, and current economic status, which was observed to affect disaster-related mental health recovery. As a result, disaster-related loss of employment (odds ratio (OR): 0.75, 95% confidence interval: 0.63–0.89) and economic hardship (OR: 0.70, 95% CI: 0.59–0.82) were associated with non-recovered mental health status. In Model 2, which had current health status, lifestyle, and social network variables added on, good general subjective health status (OR: 1.47, 95% CI: 1.20–1.80), increased or unchanged physical activity level (OR: 1.23, 95% CI: 1.01–1.50), social interaction with friends from pre-disaster (OR: 1.25, 95% CI: 1.00–1.55), and social roles through daily activities (OR: 1.44, 95% CI: 1.14–1.82) were significantly associated with mental health recovery. However, disaster-related loss of employment (OR: 0.80, 95% CI: 0.65–0.99) and economic hardship (OR: 0.80, 95% CI: 0.65–0.98) were still associated with non-recovered mental health status even when the positive effects of good general subjective health status, regular physical activity, social interaction with friends from pre-disaster, and social roles through daily activities on their mental health were considered (Table 5).

## 4. Discussion

The present study aimed to clarify the related factors associated with mental health recovery among the evacuees and residents. We consequently found that good general subjective health status, regular physical activity, and social networking (interacting with friends from pre-disaster and social roles through daily activities) were significantly associated with mental health recovery. In contrast, disaster-related loss of employment and economic hardship negatively affected mental health recovery in Fukushima evacuees and residents.

### 4.1. The Mental Health Recovery Patterns and Their Basic Characteristics

In this study, we utilized the mental health recovery pattern based on the trajectory models of resilience, recovery, and delayed/chronic disfunction presented by Norris et al. While the patterns in our study have not been validated yet, the mean K6 score in Resilience, Recovery, and Remitting were much lower than those in Delayed/Chronic dysfunction. Moreover, the mean K6 score in the “Recovered” group was significantly lower than that of the “Non-recovered” group. Thus, our patterns of mental health status changes and categorization of mental health recovery (Recovered/Non-recovered group) may be reliable to a certain extent.

Unemployed respondents were likely to be in the “Non-recovered group” in this study, suggesting an effect of employment status on their mental health recovery. In contrast, age, gender, education, and living with family members were not significantly different between the “Recovered” and “Non-recovered” groups.

In this study, the proportion of “Recovered” mental health status between the evacuation (80.0%) and non-evacuation area (84.4%) were similar. Importantly, our findings showed that even evacuees who have been forced to relocate to the outside of the evacuation area could recover their mental health status equally well when compared to the residents living in the non-evacuation area. In contrary, even some of the residents in the non-evacuation area have yet to recover their mental health status. In fact, a previous study reported that residents in the non-evacuation area had radiation anxiety and psychological distress regardless of the environmental radiation levels [43]. Moreover, some residents in the non-evacuation area had voluntarily evacuated outside of Fukushima Prefecture due to anxiety of radiation exposure. Indeed, 15.6% of residents in the non-evacuation area in this study had experienced evacuation due to a nuclear disaster (Voluntary evacuation, *n* = 27). Detailed analysis among the residents in the non-evacuation area showed that they had disaster-related experiences or economic hardship although it was lower than that of the evacuees in the evacuation area (Separated from family members: 8.7%, Disaster-associated loss of employment: 11.6%, Economic hardship: 41.3%) (Data in Supplementary Table S1). These situations may affect why there was no significant difference in the mental health recovery between evacuees in the evacuation area and the residents in the non-evacuation area (Table 2). Thus, we analyzed the association between mental health recovery and related factors in evacuees in the evacuation area and residents in the non-evacuation area as a whole as some of the residents in the non-evacuation area had some disaster-related experiences or economic hardship, and consequently could have psychological distress.

### 4.2. Association between Mental Health Recovery and Disaster-Related Experiences, Current Economic and Health Status, and Lifestyle and Social Network

In analyzing related factors associated with mental health recovery, Model 1 was adjusted by disaster-related experiences and current economic status consequent to the disaster that could cause a negative psychological reaction. In contrast, Model 2 was adjusted by health status, lifestyle, and social network, which have been provided as information or service as disaster health activities and could have positive psychological effects. Our findings in Model 1 showed that disaster-related loss of employment and economic hardship could hinder mental health recovery, and were more likely to be risk factors compared to the residential area (evacuation/non-evacuation area), or loss of family, relatives, or friends. Disaster-related loss of employment and economic hardship was an obviously stressful event and could be considered the role of relative poverty, social isolation, and decreasing opportunities for health-related behaviors [44]. This may affect the association with the hindrance of mental health recovery.

In contrast, good general subjective health condition and steady physical activities may have promoted mental health recovery among the evacuees and residents. A previous study reported that employees in the evacuation area who had good general subjective health and regular physical activity could maintain their mental health in the post-disaster period even if their work and life circumstances had significantly changed [39]. In the post disaster period of the Great East Japan Earthquake and Fukushima Daiichi nuclear power plant accident, numerous supports including maintaining the general health and physical activity level among the evacuees and residents have been received health guidance as a part of the disaster health activities [41,42]. Leisure activities including physical activities could play a role in benefitting overall well-being coping to adequately deal with stress [45,46]. Similar to the previous study, our findings showed that health habits that promoted a good general health status and physical activity could have helped to promote mental health recovery while providing health guidance at the disaster health activities.

Moreover, building a social network or social ties was also an important role of disaster health activities as many evacuees had to relocate outside of their hometown, consequently leading to separation from their original community. In fact, previous studies have reported that perceived social network is associated with reduced psychological distress or has a positive effect on mental health [47,48,49], and numerous events have been implemented in the Fukushima prefecture, particularly in the evacuation area (e.g., exchange meetings, parties in temporary housing, active listening for evacuees or residents) to help build social networks or social ties [50,51,52,53]. Our findings could indicate that these measures may have enabled social interaction with friends from pre-disaster, which consequently might promote mental health recovery.

Evacuees and residents in Fukushima were given numerous types of support from outside supporters or volunteers in the short- to mid-term in the post-disaster period. However, for evacuees and residents “to feel that they can be helpful to others through jobs, housework, or social activities”, both passive support and playing any social roles through their daily activities could promote mental health recovery during the past seven years since the disaster. A previous study reported that social roles had a significant positive effect on mental health and their quality might help prevent depression or anxiety disorders [54]. Indeed, the loss of employment was a risk factor for mental health recovery in this study, with concomitant loss of opportunities for activities that could enable them to feel helpful to others. In short, our findings suggest that any social role that enables one to feel helpful to others might be related to mental health recovery in the long-term following a disaster.

Our findings showed that disaster-related loss of employment and economic hardship were still associated with non-recovered mental health status even after adjusting for other health and social network factors that positively affected mental health recovery. A previous study showed that socio-economic issues were strongly and significantly associated with the needs of long-term disaster mental health support [55]. Moreover, disaster-induced socioeconomic changes were associated with poor subjective health even after adjusting for lifestyle-related factors such as sleep, community participation, or regular exercise [35]. Thus, our findings provide insights for disaster mental health service providers, where health and socio-economic support is essential for evacuees and residents to recover their mental health status in the long-term following a disaster.

Meanwhile, the three variables that were significantly associated with mental health recovery in a univariate analysis (loss of family, relatives or friends, sleep satisfaction, and frequency of laughing) lost a significance in the multivariable analysis. The reason might be influenced by adjusting covariates (i.e., age, gender or Evacuation/Non-evacuation area).

### 4.3. Limitations and Strengths

This study had several limitations. First, causality could not be established due to its cross-sectional design. Second, the mental health recovery patterns that we utilized in this study were not validated measurements. Moreover, there was no clear definition of the six-patterns because participants selected the most appropriate mental health status changes subjectively. Third, the response rate was less than 50%, and previous studies have reported its correlation with mental health status, which suggests non-response as a consequence to poor mental health status [56]. Many evacuees in poor condition may not have wanted to or been able to answer the survey, thus leading to an underestimation in our analysis. Fourth, recall bias should be considered because we conducted this survey after the disaster and by self-reporting. Finally, the measurements for disaster-related experience, economic status, general subjective health condition, lifestyle, and social network in this study were non-validated and subjective. Therefore, caution is necessary in interpreting the findings.

## 5. Conclusions

Despite these limitations, we were able to show a positive association between mental health recovery and good general subjective health condition, regular physical activities, and social networking. In particular, the provision of passive supports and any social roles enabling one to feel helpful to others could promote mental health recovery. Despite these, disaster-related loss of employment and economic hardship still hindered mental health recovery, indicating the necessity of socio-economic support for evacuees and residents in addition to health support. Our findings could potentially aid in preparing to support evacuees in future disasters.

## Figures and Tables

**Table 1:** Basic characteristics of respondents (each mental health recovery patterns).

Basic Characteristics	Total		Pattern 1		Pattern 2		Pattern 3		Pattern 4		Pattern 5		Pattern 6		None of These Patterns	
			Resistance		Resilience		Recovery		Remitting		Delayed Dysfunction		Chronic Dysfunction			
	(*n* = 410)		(*n* = 42)		(*n* = 100)		(*n* = 127)		(*n* = 47)		(*n* = 19)		(*n* = 40)		(*n* = 35)	
	*n* (%)		*n* (%)		*n* (%)		*n* (%)		*n* (%)		*n* (%)		*n* (%)		*n* (%)	
Age (as of February 2018)																
Less than 40 years old	56	(100.0)	9	(16.1)	9	(16.1)	15	(26.8)	12	(21.4)	6	(10.7)	1	(1.8)	4	(7.1)
40–64 years old	190	(100.0)	19	(10.0)	50	(26.3)	58	(30.5)	22	(11.6)	7	(3.7)	17	(8.9)	17	(8.9)
65 years old and more	164	(100.0)	14	(8.5)	41	(25.0)	54	(32.9)	13	(7.9)	6	(3.7)	22	(13.4)	14	(8.5)
Gender																
Male	178	(100.0)	27	(15.2)	42	(23.6)	51	(28.7)	14	(7.9)	9	(5.1)	18	(10.1)	17	(9.6)
Female	232	(100.0)	15	(6.5)	58	(25.0)	76	(32.8)	33	(14.2)	10	(4.3)	22	(9.5)	18	(7.8)
Education																
Junior/Senior high school	300	(100.0)	23	(7.7)	67	(22.3)	100	(33.3)	35	(11.7)	14	(4.7)	33	(11.0)	28	(9.3)
Vocational college, University	108	(100.0)	19	(17.6)	33	(30.6)	26	(24.1)	11	(10.2)	5	(4.6)	7	(6.5)	7	(6.5)
Occupational category																
Employed, Owner	154	(100.0)	24	(15.6)	44	(28.6)	42	(27.3)	19	(12.3)	4	(2.6)	11	(7.1)	10	(6.5)
Part-time	49	(100.0)	1	(2.0)	13	(26.5)	16	(32.7)	8	(16.3)	2	(4.1)	3	(6.1)	6	(12.2)
Homemaker	77	(100.0)	8	(10.4)	19	(24.7)	26	(33.8)	12	(15.6)	1	(1.3)	6	(7.8)	5	(6.5)
Unemployed	122	(100.0)	9	(7.4)	20	(16.4)	41	(33.6)	7	(5.7)	11	(9.0)	20	(16.4)	14	(11.5)
Living area as of 11 March 2011																
Evacuation area	177	(100.0)	8	(4.5)	30	(16.9)	72	(40.7)	26	(14.7)	8	(4.5)	24	(13.6)	9	(5.1)
Non-evacuation area	233	(100.0)	34	(14.6)	70	(30.0)	55	(23.6)	21	(9.0)	11	(4.7)	16	(6.9)	26	(11.2)
Living with family member																
Living with family	362	(100.0)	40	(11.0)	93	(25.7)	111	(30.7)	39	(10.8)	14	(3.9)	35	(9.7)	30	(8.3)
Single life	38	(100.0)	1	(2.6)	7	(18.4)	14	(36.8)	7	(18.4)	3	(7.9)	3	(7.9)	3	(7.9)
Current psychological distress (K6: Kessler 6)																
K6 score ≥ 13	50	(100.0)	5	(12.8)	4	(4.0)	3	(2.5)	6	(13.6)	7	(36.8)	20	(54.1)	5	(14.7)
K6 score (mean, SD)	6.0	(5.3)	5.2	(5.7)	3.1	(3.9)	5.5	(3.7)	6.8	(4.4)	11.8	(5.1)	12.3	(5.4)	6.4	(6.38)

**Table 2:** Basic characteristics of subjects (Recovered/Non-recovered group).

Basic Characteristics	Total		Recovered		Non-Recovered		*p*-Value (χ^2^/t)
	(*n* = 333)		(*n* = 274)		(*n* = 59)		
	*n* (%)		*n* (%)		*n* (%)		
Age (as of February 2018)							
Less than 40 years old	43	(100.0)	36	(83.7)	7	(16.3)	
40–64 years old	154	(100.0)	130	(84.4)	24	(15.6)	0.52 (χ^2^ = 1.31)
65 years old and more	136	(100.0)	108	(79.4)	28	(20.6)	
Gender							
Male	134	(100.0)	107	(79.9)	27	(20.1)	0.34 (χ^2^ = 0.91)
Female	199	(100.0)	167	(83.9)	32	(16.1)	
Education							
Junior/Senior high school	249	(100.0)	202	(81.1)	47	(18.9)	0.38 (χ^2^ = 0.76)
Vocational college, University	82	(100.0)	70	(85.4)	12	(14.6)	
Occupational category							
Employed, Owner	120	(100.0)	105	(87.5)	15	(12.5)	
Part-time	42	(100.0)	37	(88.1)	5	(11.9)	
Homemaker	64	(100.0)	57	(89.1)	7	(10.9)	<0.01 (χ^2^ = 19.5)
Unemployed	99	(100.0)	68	(68.7)	31	(31.3)	
Living area as of 11 March 2011							
Evacuation area	160	(100.0)	128	(80.0)	32	(20.0)	0.29 (χ^2^ = 1.10)
Non-evacuation area	173	(100.0)	146	(84.4)	27	(15.6)	
Living with family member							0.90 (χ^2^ = 0.02)
Living with family	292	(100.0)	243	(83.2)	49	(16.8)	
Single life	34	(100.0)	28	(82.4)	6	(17.6)	
Current psychological distress (K6: Kessler6)							
K6 score ≥ 13	40	(100.0)	13	(32.5)	27	(67.5)	<0.01 (χ^2^ = 79.2)
K6 score ≤ 12	280	(100.0)	251	(89.6)	29	(10.4)	
K6 score (mean, SD)	6.0	(5.3)	4.8	(4.1)	12.1	(5.2)	<0.01 (*t* = 9.81)

K6 score was tested by the *t*-test. The others were tested by χ^2^ test. SD: Standard Deviation.

**Table 3:** Disaster-related experience and current economic status.

Disaster-Related Experience and Current Economic Status	Total		Recovered		Non-Recovered		*p*-Value (χ^2^)
	(*n* = 333)		(*n* = 274)		(*n* = 59)		
	*n* (%)		*n* (%)		*n* (%)		
Evacuation							
Experienced	176	(100.0)	144	(81.8)	32	(18.2)	0.82 (χ^2^ = 0.06)
Never	157	(100.0)	130	(82.8)	27	(17.2)	
Separation from family members							
Experienced	103	(100.0)	82	(79.6)	21	(20.4)	0.39 (χ^2^ = 0.73)
Never	230	(100.0)	192	(83.5)	38	(16.5)	
House damage (severe/partial collapse)							
Experienced	117	(100.0)	93	(79.5)	24	(20.5)	0.33 (χ^2^ = 0.97)
Never	216	(100.0)	181	(83.8)	35	(16.2)	
Loss of family, relatives or friends							
Experienced	59	(100.0)	41	(69.5)	18	(30.5)	0.01 (χ^2^ = 8.05)
Never	274	(100.0)	233	(85.0)	41	(15.0)	
Disaster-related loss of employment							<0.01 (χ^2^ = 17.9)
Experienced	81	(100.0)	54	(66.7)	27	(33.3)	
Never	252	(100.0)	220	(87.3)	32	(12.7)	
Economic status (Afford to live in current economic status)							
Difficult	110	(100.0)	76	(69.1)	34	(30.9)	<0.01 (χ^2^ = 20.4)
Enough/Average	221	(100.0)	197	(89.1)	24	(10.9)	

**Table 4:** Current health status, lifestyle, and social status.

Current Health Status, Lifestyle and Social Status	Total		Recovered		Non-Recovered		*p*-Value (χ^2^)
	(*n* = 333)		(*n* = 274)		(*n* = 59)		
	*n* (%)		*n* (%)		*n* (%)		
General subjective health status							
Very well/Well/Unremarkable	249	(100.0)	228	(91.6)	21	(8.4)	<0.01 (χ^2^ = 61.6)
Poor/Very poor	81	(100.0)	43	(53.1)	38	(46.9)	
Sleep condition							
Satisfied with sleep condition	149	(100.0)	137	(91.9)	12	(8.1)	<0.01 (χ^2^ = 17.3)
Dissatisfied	184	(100.0)	137	(74.5)	47	(25.5)	
Changes in physical activities							
Increase/No change	203	(100.0)	182	(89.7)	21	(10.3)	<0.01 (χ^2^ = 19.7)
Decrease	125	(100.0)	88	(70.4)	37	(29.6)	
Changes in alcohol consumption							
Increase	42	(100.0)	33	(78.6)	9	(21.4)	
No change	111	(100.0)	95	(85.6)	16	(14.4)	0.22 (χ^2^ = 4.47)
Decrease	42	(100.0)	31	(73.8)	11	(26.2)	
Non-drinker	109	(100.0)	94	(86.2)	15	(13.8)	
Frequency of laughing							
Almost everyday	80	(100.0)	76	(95.0)	4	(5.0)	<0.01 (χ^2^ = 11.7)
Less that 1–5 times/week	253	(100.0)	198	(78.3)	55	(21.7)	
Social network status							
Social interaction with friends from pre-disaster							
Agree	190	(100.0)	173	(91.1)	17	(8.9)	<0.01 (χ^2^ = 16.1)
Disagree/Neither or not	132	(100.0)	94	(71.2)	38	(28.8)	
Place to communicate about the disaster							
Agree	119	(100.0)	104	(87.4)	15	(12.6)	0.06 (χ^2^ = 3.59)
Disagree/Neither or not	205	(100.0)	162	(79.0)	43	(21.0)	
Social roles through daily activities							
Agree	138	(100.0)	129	(93.5)	9	(6.5)	<0.01 (χ^2^ = 21.4)
Disagree/Neither or not	189	(100.0)	139	(73.5)	50	(26.5)	

**Table 5:** Multivariable logistic regression analysis between mental health recovery and related factors.

Relative Factors with Mental Health Recovery			Model 1 (*n* = 331)			Model 2 (*n* = 310)		
			OR (95% CI)		*p*-Value	OR (95% CI)		*p*-Value
Basic characteristics								
Age			1.02	(0.99–1.04)	0.15	1.00	(0.98–1.03)	0.78
Gender		Male	0.72	(0.39–1.35)	0.31	0.72	(0.33–1.57)	0.41
		Female (Ref.)	1.00			1.00		
Living area as of 11 March 2011		Evacuation	0.97	(0.82–1.16)	0.77	0.97	(0.78–1.22)	0.82
		non-evacuation (Ref.)	1.00			1.00		
Disaster-related experience								
Loss of family, relatives or friends		Experienced	0.84	(0.70–1.02)	0.08	0.89	(0.71–1.13)	0.34
		Never (Ref.)	1.00			1.00		
Disaster-related loss of employment		Experienced	**0.75**	**(0.63–0.89)**	**<0.01**	**0.80**	**(0.65–0.99)**	**0.04**
		Never (Ref.)	1.00			1.00		
Economic status								
Afford to live in current economic status		Difficult	**0.70**	**(0.59–0.82)**	**<0.01**	**0.80**	**(0.65–0.98)**	**0.03**
		Enough/Average (Ref.)	1.00			1.00		
Current health status and lifestyle								
General subjective health status		Well/Unremarkable				**1.47**	**(1.20–1.80)**	**<0.01**
		Poor/Very poor (Ref.)				1.00		
Sleep condition		Satisfied with sleep				1.09	(0.86–1.37)	0.49
		Dissatisfied (Ref.)				1.00		
Changes in physical activities		Increase/No change				**1.23**	**(1.01–1.50)**	**0.04**
		Decrease (Ref.)				1.00		
Frequency of laughing		Almost everyday				1.19	(0.85–1.67)	0.30
		Less that 1–5 times/week (Ref.)				1.00		
Social network status								
Social interaction with friends		Agree				**1.25**	**(1.00–1.55)**	**0.05**
		Disagree/Neither or not (Ref.)				1.00		
Place to communicate about the disaster		Agree				0.81	(0.63–1.03)	0.08
		Disagree/Neither or not (Ref.)				1.00		
Social roles through daily activities		Agree				**1.44**	**(1.14–1.82)**	**<0.01**
		Disagree/Neither or not (Ref.)				1.00		

**OR**: Odds Ratio, **CI**: Confidence Interval, **Bold**: *p* < 0.05. **Model 1**: Adjusted by age, gender, disaster-related experiences, and current economic status variables. **Model 2**: Adjusted current health status and lifestyle, and social network status added on to the variables in Model 1.

## Supplementary Materials

The following are available online at http://www.mdpi.com/1660-4601/15/11/2381/s1, Table S1: Disaster-related experience and current economic status (Evacuation/Non-evacuation area).
