# Supplementary material for: Mental Health Recovery of Evacuees and Residents from the Fukushima Daiichi Nuclear Power Plant Accident after Seven Years—Contribution of Social Network and a Desirable Lifestyle
Source: Int J Environ Res Public Health. 2018 Oct 27;15(11):2381. doi: 10.3390/ijerph15112381 (PMC6265751; doi:10.3390/ijerph15112381)
Supplement: Supplementary file 1 [file ijerph-15-02381-s001.pdf]

Supplementary

Mental Health Recovery of Evacuees and Residents from the Fukushima Daiichi Nuclear Power Plant Accident after Seven Years—Contribution of Social Network and a Desirable Lifestyle

Masatsugu Orui, Satomi Nakajima, Yui Takebayashi, Akiko Ito, Maho Momoi, Masaharu Maeda, Seiji Yasumura and Hitoshi Ohto

Table S1. Disaster-related experience and current economic status (Evacuation/Non-evacuation area)

	**Total**		**Evacuees in evacuation area**		**Residents in non-evacuation area**		
	(n=333)		(n=160)		(n=173)		p-value
	n (%)		n (%)		n (%)		(χ^2)
Evacuation							
Experienced	176	(52.9)	149	(93.1)	27	(15.6)	<0.01
Never	157	(47.1)	11	(6.9)	146	(84.4)	(χ^2=200.4)
Separation of family members due to a nuclear disaster							
Experienced	119	(35.7)	88	(55.0)	15	(8.7)	<0.01
Never	230	(69.1)	72	(45.0)	158	(91.3)	(χ^2=83.5)
House damage (severe/partial collapse)							
Experienced	117	(35.1)	69	(43.1)	48	(27.7)	<0.01
Never	216	(64.9)	91	(56.9)	125	(72.3)	(χ^2=8.63)
Loss of family, relatives or friends							
Experienced	59	(17.7)	46	(28.8)	13	(7.5)	<0.01
Never	274	(82.3)	114	(71.3)	160	(92.5)	(χ^2=25.7)
Loss of employment due to disaster							
Experienced	81	(24.3)	61	(38.1)	20	(11.6)	<0.01
Never	252	(75.7)	99	(61.9)	153	(88.4)	(χ^2=31.9)
Economic status (Afford to live in current economic status)							
Difficult	110	(33.2)	39	(24.5)	71	(41.3)	<0.01
Enough/ Average	221	(66.8)	120	(75.5)	101	(58.7)	(χ^2=10.4)

© 2018 by the authors. Submitted for possible open access publication under the terms and conditions of the Creative Commons Attribution (CC BY) license (http://creativecommons.org/licenses/by/4.0/).
